# Influence of surgical ultrasound used in the detachment of flaps, osteotomy and odontosection in lower third molar surgeries. A prospective, randomized, and “split-mouth” clinical study

**DOI:** 10.4317/medoral.23447

**Published:** 2020-05-10

**Authors:** Leonardo de Freitas Silva, Erik Neiva Ribeiro de Carvalho Reis, João Paulo Bonardi, Valthierre Nunes de Lima, Alessandra Marcondes Aranega, Daniela Ponzoni

**Affiliations:** 1 MD, Doctorate in Dentistry, Buccomaxillofacial Surgery and Traumatology, Department of Surgery and Integrated Clinic, Araçatuba School of Dentistry - UNESP, São Paulo, Brazil; 2 MD, PhD, Assistant Professor, Department of Surgery and Integrated Clinic, Araçatuba School of Dentistry - UNESP, São Paulo, Brazil

## Abstract

**Background:**

As third molar surgery is the most commonly procedure performed in Dentistry and has been accompanied by serious postoperative disorders such as pain, edema and trismus, the study aimed to evaluate if ultrasound device would be able to reduce such postoperative features. The aim of this study was to assess the effects of soft tissue flap elevation, osteotomy and odontosection using piezosurgery versus conventional technique in mandibular third molar extractions.

**Material and Methods:**

Twenty patients with impacted mandibular third molars underwent tooth extractions using two different methods. Ten patients were included in the Piezo Flap Group (PFG - the flap was elevated using piezosurgery) and ten patients were part of the Piezo Ostectomy Group (POG - osteotomy and odontosection were carried out with ultrasound tips). The contralateral tooth was included in the Control Group (CG - conventional technique). The patients were evaluated at postoperative periods of 1, 3, 7 and 14-days. The measured parameters were duration of surgery, pain, trismus and swelling.

**Results:**

The mean duration of surgery for the PFG was 17.21 minutes (CG 10.07 minutes) and POG was 40.09 minutes (CG 15.97 minutes). There was no statistically significant difference in pain and trismus for any of the postoperative periods evaluated in PFG and POG (*p*>0.05). There was a statistically significant difference in swelling between the PFG and POG, presenting less swelling at the 3-day postoperative period (*p*=0.038; *p*<0,05). However, for the remaining analyzed periods there was no difference (*p*>0.05).

**Conclusions:**

Piezosurgery for tissue elevation of the surgical flap, osteotomy and dental sectioning in mandibular third molar extraction surgery promoted less edema in the early postoperative stages in mandibular third molar extractions despite the longer surgical duration.

** Key words:**Third molar, piezosurgery, flap, exodontia, ostectomy.

## Introduction

Mandibular third molar extraction is one of the most common procedures carried out by oral and maxillofacial surgeons (1,2). This surgery involves soft tissue incisions, ostectomies, and tooth sectioning and often results in postoperative complications such as pain, swelling, and trismus (3).

The piezoelectric device uses ultrasonic micro-vibrations that cut hard tissue while preserving soft tissue (4,5). The device’s frequency is usually adjusted to between 25 and 30 kHz. This frequency generates micro-vibrations that are 60 to 210 μm wide, providing the device with a power that is greater than 5 W (4,5). Ultrasound has been used successfully in endodontic surgery, implantology, orthognathic surgery, reconstructive surgery, otologic surgery, orthopedics and neurosurgery (4-7).

Other studies have shown that ultrasound for bone surgery is precise and safe and does not cause bleeding within the surgical field. For maxillary sinus lift surgery, ultrasound techniques reduced the Schneiderian membrane perforation risk, accelerated bone regeneration, and decreased postoperative pain (8). Goyal *et al*. (9) and *Pi*ersanti *et al*. (10) used piezosurgery to extract inferior third molars and observed a reduction in pain and swelling when compared with conventional technique (9,10).

The greatest disadvantage of ultrasound use in bone surgery is the increase in surgical time (8). Goyal *et al*. (9) and Rullo *et al*. (11), reported significantly greater operative time for inferior third molar extraction with piezosurgery compared with conventional technique (9,11).

The use of piezosurgery for elevation of mucoperiosteal flaps has been suggested as an alternative technique to reduce postoperative discomfort and swelling; however, this suggestion was made without a sound scientific basis. Thus, the primary motive for this study is to provide a sound scientific basis.

The need for development of minimally traumatic surgical procedures in oral surgery has led to the evolution of technological advancements such as piezosurgery.

The aim of this study was to assess the effects of piezosurgery on soft tissue flap elevation, osteotomy and odontectomy, during the postoperative period of inferior third molar extraction.

The null hypothesis tested was that piezosurgery used in flap elevation, osteotomy and odontectomy would not bring benefits on the postoperative period of inferior third molar extraction compared to a conventional technique.

## Material and Methods

All patients who took part in the study signed an authorization and disclosure consent form following the medical and ethical protocols of Helsinki statement, 2013 (12).

- Study design

Twenty patients were included in this study. The website http:www.lee.dante.br was used for the sample size calculation, which substantiates in previous results (10). The standard-deviation used was 0.23, the difference of means was 0.35, the power of the test was 80% and *p*<0.05, with five patients per group. Therefore, for a split-mouth study, ten patients per group were selected for this study with two sites of analysis per patient (*Pi*ezo Flap/ *Pi*ezo Ostectomy Group versus Control Group).

 All patients presented with bilateral mandibular third molars that were either semi or totally impacted, indicating the need for extraction through bone removal and dental segmentation. The third molars should be buried or partially buried in mandibular bone, in position B or C (below the oclusal plane of second molar) and mesioangular (mesially leaned in relation to second molar axis) according Pell and Gregory’s classification (13). Panoramic radiographs were used to confirm that the teeth of both sides in each patient had a similar level of surgical difficulty.

Smokers, patients with periodontal disease or uncontrolled systemic diseases, and patients using medication were excluded from the study.

Each patient had both mandibular third molars removed in two different surgeries. The interval between the two surgeries was at least 15 days. All surgeries were carried out by the same experienced surgeon.

In each patient, one of the teeth was included in the Control group (conventional technique) and the other tooth was included in the *Pi*ezo Flap Group (flap elevation with ultrasound tip – 10 teeth) or in the *Pi*ezo Osteotomy Group (osteotomy and odontectomy performed with surgical ultrasound – 10 teeth). The selection of the side of the mouth to be used in each group was randomized through systematic randomization. A third researcher present in the operative room was responsible for taking the paper from the envelopes (first “Control” or “*Pi*ezo”; then “*Pi*ezo Flap” or “*Pi*ezo Osteotomy”). The examiner was blinded regarding the surgical method.

The dependent variables in this study were the technique of flap elevation: manual flap elevation with a Molt detacher or with the surgical ultrasound device; and cutting of the hard tissues: use of high-speed burr or the surgical ultrasound.

The measured parameters were duration of surgery, pain, swelling, trismus, and dehiscence in the postoperative period. The patients were evaluated preoperatively and at the postoperative periods of 1, 3, 7, and 14-days. The examiner and surgeon were different individuals.

- Surgical procedure

After intra and extra oral antisepsis, the patients received anesthesia consisting of the inferior alveolar, lingual and buccal nerves block technique using mepivacaine 2% with 1:100.000 of adrenaline (Mepiadre Nova DFL®, Rio de Janeiro, Rio de Janeiro, Brazil).

In the Control group, a number 15 surgical scalpel blade was used for the incision, a number 9 Molt elevator (Quinelato®, Rio Claro, São Paulo, Brazil) for the flap elevation, and a number 702 burr (KG Sorensen®, Cotia, São Paulo, Brazil) connected to a handpiece (KaVo do Brazil Ind. Com. Ltd, Joinville, Santa Catarina, Brazil) for removal of the bone and sectioning of the tooth. The site was abundantly irrigated with sterile saline solution throughout surgery.

**Figure 1:**
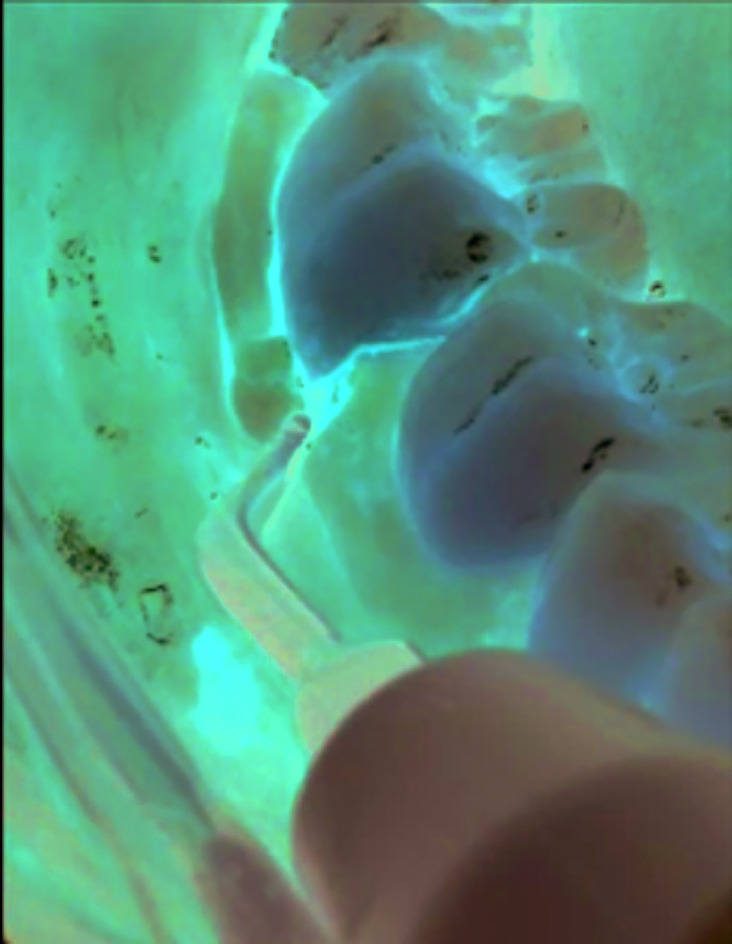
*Pi*ezo Flap Group. Flap detachment with surgical ultrasound.

In the *Pi*ezo Flap Group, the flap elevation was carried out using surgical ultrasound (Fig. 1) and the surgical sequence was like the control group.

**Figure 2:**
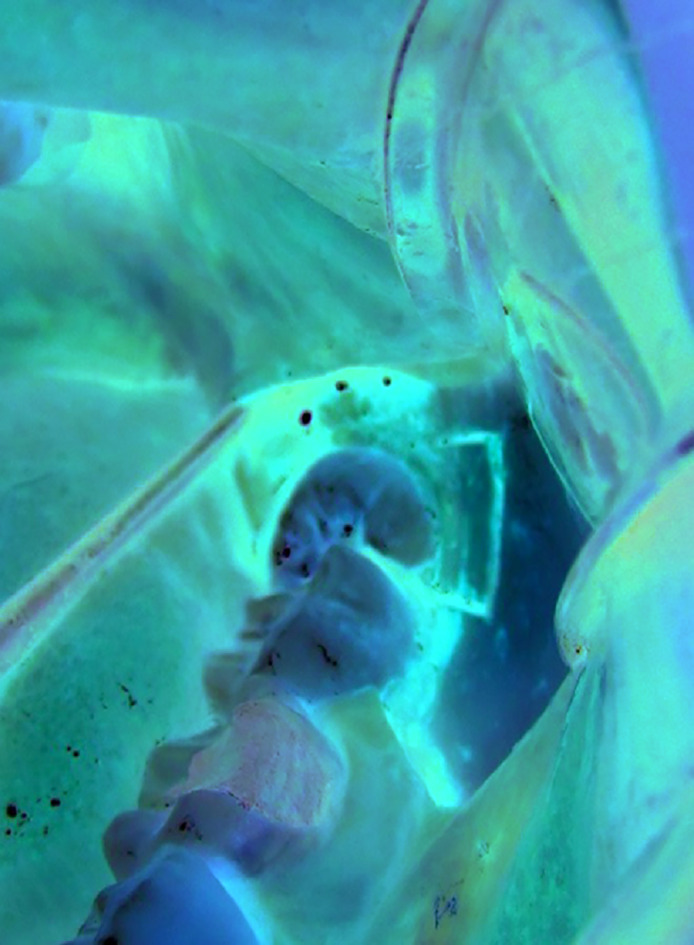
*Pi*ezo Osteotomy Group. Osteotomy and odontectomy were carried out with surgical ultrasound.

In the *Pi*ezo Osteotomy Group, the osteotomy and odontectomy were carried out with surgical ultrasound (*Pi*ezosonic, Driller®, Carapicuíba, São Paulo, Brazil). The osteotomy was carried out in a rectangular shape using the ES007R and ES007L tips (Fig. 2) with the objective of partial removal of the buccal bone cortex. The bone block was removed with a tissue detacher [9,20]. The odontectomy was carried out with the tip ESOO9. The other steps of the extraction were performed in a similar way to the Control group.

The teeth were removed using a dental extractor and the surgical wound was sutured with wire silk 4-0 (Ethicon®, Johnson & Johnson, São Paulo, São Paulo, Brazil) using simple sutures.

After surgeries patients received postoperative antibiotics (Amoxicillin 500 mg Medley®, Campinas, São Paulo, Brazil) orally, every 8 hours for 5 days, a non-steroidal anti-inflammatory (Nimesulide 100 mg Medley®, Campinas, São Paulo, Brazil) orally every 12 hours for 3 days, and an analgesic (Dypirone sodium 500 mg Medley®, Campinas, São Paulo, Brazil) orally every 6 hours for pain. The sutures were removed seven days after surgery.

- Analysis of the variables

The duration of surgery was defined as the time interval (in minutes) between the incision and removal of the third molar.

Pain was evaluated using the visual analog scale, with the following scores: 0 (absence of pain), 1-3 (low pain), 4-6 (moderate pain), 7-9 (severe pain), 10 (worst pain) (14). For the data analysis each score received a new score to facilitate the statistical analysis. Thus, 0 score was considered 1 score; 1-3 variation was considered 2 score; 4-6 variation was considered 3 score; 7-9 variation was considered 4 score and, 10 score was considered 5 score. The pain data representation was performed through mean of these new scores among patients.

Swelling was assessed by taking horizontal and vertical measurements of the face before surgery and during the postoperative periods of 1, 3, 7, and 14-days. The horizontal measurement was the distance from the labial commissure to the ipsilateral ear insertion. The vertical measurement was from the external canthus of the eye to the mandibular angle (located by manually touching the inferior border of the mandible) (14).

The measurements were initially obtained using a Nylon wire 3-0 (Technofio®, Goiânia, Goiás, Brazil) and the length calculated by placing the wire against a standard metric ruler. The facial measurements were calculated in millimeters and expressed as the simple mean between the vertical and horizontal measurements (12). The percentage of facial edema was calculated according to the following equation: (postoperative measurement – preoperative measurement / preoperative measurement × 100) (14).

Trismus was assessed by the measurement of the maximum mouth opening, using an analog pachymeter, between the incisal borders of the upper and lower central incisors. Trismus was calculated according to the following equation: (preoperative measurement – postoperative measurement / preoperative measurement X 100) (14).

The presence of dehiscence of the surgical wound was evaluated seven days after surgery. Dehiscence was defined as the presence of a tissue gap at the incision site (12).

Additionally, the patient’s preference for each method was recorded.

- Statistical analysis

Data were tabulated and compared statistically in the statistical program SigmaPlotTM 12.3 (SigmaPlot Exakt Graphs and Data Analysis, San Jose, CA). The data were compared by Shapiro‒Wilk homoscedasticity test, which shows homogeneity. Analysis of variance (two-factor ANOVA) and post-hoc Tukey test were applied. For all tests, the level of 5% was considered significant.

## Results

Twenty patients (17 female and 3 male) ranging in age from 16 to 37 years, participated in this study. Ten patients presented vertical third molars, four presented mesio-angular molars, four presented horizontal molars and two presented disto-angular molars.

The mean duration was 13,02 minutes for the Control Group (20 teeth), 17.21 minutes for the *Pi*ezo Flap Group (10 teeth) and 40.09 minutes for the *Pi*ezo Osteotomy Group.

Tables show the percentage of edema, trismus and analysis of visual analog scale during the postoperative periods of each Group. Dehiscence or any other serious postoperative complications, such as infections, alveolitis, paraesthesia, were not observed in any of the individuals.

With regards to the method used, seven patients preferred the conventional surgery and the other three patients preferred the piezosurgery.

- Statistical analysis

The percentage of swelling showed a significant decrease between the initial postoperative periods (1 to 3 days) and final postoperative periods (7 to 14 days). When the two study groups were compared three days after surgery, the swelling in the *Pi*ezo Flap Group and *Pi*ezo Osteotomy Group was statistically significantly less than the Control group (*p*=0.038 and *p*<0.001). In remaining postoperative periods (1, 7 and 14 days) there was no statistical difference between the groups (Table 1).

Trismus was not significantly different between groups in the postoperative periods (Table 2). However, the reduction of trismus was significant overall during the postoperative period when compared with the initial time interval (1 to 3 days) and final interval (7 to 14 days).

In all groups, there was a reduction in pain scores with the increase of the postoperative time. All groups presented a significant pain score reduction (*p*<0.001) when comparing one and three days after surgery with 14 days after surgery. The pain scores between each postoperative evaluation interval (1 to 3, 3 to 7 and 7 to 14 days) were not significantly different. There was no significant difference between the groups in all observed postoperative periods (Table 3).

**Table 1:**
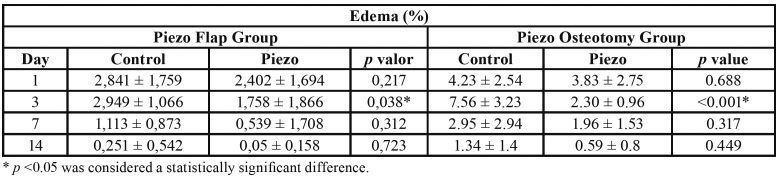
Analysis of edema comparisons between groups according to observation periods.

**Table 2:**
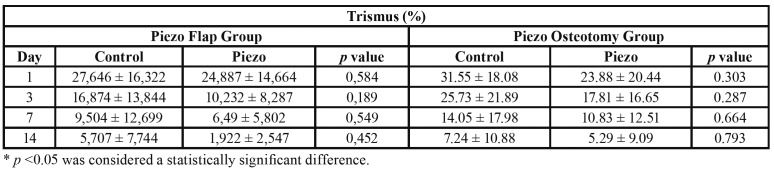
Analysis of trismus comparisons between groups according to observation periods.

**Table 3:**
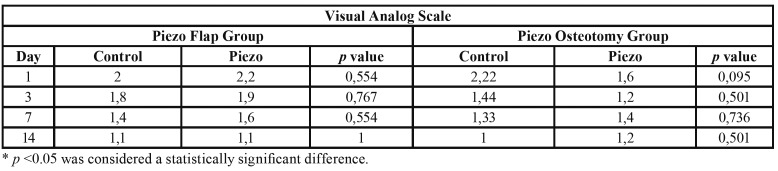
Analysis of visual analog scale comparisons between groups according to observation periods.

## Discussion

Surgical ultrasound has been used in osteotomies to reduce tissue injury and preserve soft tissue (7). These benefits were the motivation for this study, specifically the suggestion that soft tissue is submitted to less injury during flap elevation. If the periosteum can maintain its structural integrity during detachment from cortical bone tissue, this would theoretically result in a major increase in postoperative comfort for the patient. In this study we also tested sectioning of hard tissues, osteotomy and odontectomy, with conventional flap detachment way with a Molt elevator.

One of the limitations of this study is that double blinding was not possible for obvious reasons. However, the evaluators were unaware of the surgical technique used.

The method used in this study for exodontia followed the conventional technique of anesthesia and access to the lower third molars, using a triangular incision. Sandhu *et al*. (14) analyzed two different types of incision design for the removal of impacted lower third molars and observed better postoperative results with the preparation of a buccal relaxant incision. The authors used the same postoperative parameters that were used in this study.

The surgical time of the extractions that used the ultrasound motor was significantly higher in the *Pi*ezo Ostectomy Group than Control Group (mean of 15.97 minutes for the Control Group and 40.09 minutes for the *Pi*ezo Ostectomy Group). This result corroborates other studies that mention this as the main disadvantage of the use of the ultrasound motor in third molar extractions (9,10,11). In this study, it was observed that the longest operative time was the dental sectioning with the ultrasound device due to the greater hardness of the dental tissues when compared with the bone tissue. Surgical time usually correlates with increased trismus, edema, pain and the number of analgesics taken by patients. Although the surgical time was significantly higher in the *Pi*ezo Ostectomy Group, the patients presented lower trismus, edema and intake of analgesics. The piezotome delivers a micrometric cut involving the minimum surface area; this may be one of the factors that contribute to the good results obtained. Osteotomies were done with a minimal risk of an increase in temperature and marginal osteonecrosis as a result of thermal injury. An osteotomy with a piezoelectric motor rarely causes thermal damage to the bone tissue when irrigation is maintained and there is no excessive pressure at the tips (5). It is believed that during the odontosection the piezosurgery should also have generated less heat compared to the conventional rotational one, considering that the dental tissue, enamel and dentin offer greater resistance to the cut in relation to the bone tissue, which may have improved our results.

Edema was lower in the *Pi*ezo Flap Group and in the *Pi*ezo Osteotomy Group at all observation periods and was significantly lower at 3 days (*p*=0,038; *p* <0.001). This finding is in line with the results of Al Moraissi *et al*. (15) and Magesty *et al*. (16) who carried out systematic reviews and meta-analysis on rotational instruments and piezo-surgery in the removal of third molars. In several other studies, the *Pi*ezo surgery was also of longer duration than the conventional surgery. With the use of rotating instruments, generally larger edemas are expected in the postoperative period of longer surgeries. A better postoperative was also reported by Keyhan *et al*. (17) and Patil *et al*. (18) Menziletoglu *et al*. (19), on the other hand, reports that there were no differences in the postoperative period between piezo and conventional instruments.

Swelling, trismus and postoperative pain are often present in the postoperative phase of third molar surgeries. This was consistent with what was observed in the follow-up period of both groups. At day three of the postoperative period, patients reported these symptoms as intense. The symptoms then decreased noticeably in the subsequent observation periods.

The third day after surgery is when swelling typically reaches its peak. Thus, the measurements taken of swelling at this time showed the greatest statistical difference between the Control and the *Pi*ezo Groups. At this time, swelling was significantly lower in the *Pi*ezo Groups than in the Control group, despite the longer surgery duration. The smaller edema in the *Pi*ezo Groups was probably associated with the less injury to the periosteum and and lower bone heating. Although the trismus difference between groups was not statistically significant, the *Pi*ezos Groups showed smaller values than the Control group in all follow-up periods.

There was no difference in trismus between the groups at the observation periods. The results observed in the piezo group were lower at all postoperative times compared with the control group, although not significantly lower, as also found in the study of *Pi*ersanti *et al*. (10).

Another advantage of using the piezoelectric device is the selective cutting of the hard tissues without damaging the soft tissues, which can avoid nerve damage when in close proximity with the tooth to be extracted. In addition, a better visibility of the operative field has been observed due to the hemostatic effect that the tips have on the blood vessels, unlike rotatory instruments. The oscillating tip drives the irrigation solution, which allows for better visibility and the evacuation of detritus (through the cavitation phenomenon, which is implosion of gas bullae into blood vessels during bony cutting which produces a haemostatic effect and so reduces blood loss) in the operating field, compared with conventional osteotomy burs (9).

Most patients reported a preference for the conventional method. Two factors contributed to this observation: longer surgical duration and discomfort with the noise of the ultrasound device, which was a novelty, since the high-speed handpiece sound is familiar patients. The patients who preferred the piezo method reported less edema in the postoperative period, as observed in the study of Goyal *et al*. (9). However, the study of Goyal *et al*. (9) used the ultrasound device both for the flap elevation and cutting bone and tooth sectioning.

During the operative time, there was noticeably less bleeding and better visibility of the surgical field when using the piezo method, similar to results reported by Pavlíková *et al*. (4) and Rahanama (8). This method also enabled the surgeon to keep the periosteal plane intact. Birkenfield (5) and Rahnama (8) showed that, although there was greater need of saline irrigation during piezosurgery, this technique still has other benefits, such as extreme precision, safety and selectivity of tissue divulsion.

The main disadvantages of the piezoelectric device used on hard tissues is the slow speed of cutting (concerns the increased operating time), the rupture of the surgical tips, especially when used on dental tissues, and the cost of the equipment and the ultrasonic tips, which is higher than the conventional method (9). The cost of the piezosurgery equipment is much higher than the Molt elevator and burs. However, the increased patient postoperative comfort due to reduced edema is a compelling factor for its use.

The bone density of the alveoli after extraction of the lower third molars with rotary instruments and surgical ultrasound was similar in both groups in the study of de Freitas Silva *et al*. (20).

This study concluded that using piezosurgery for tissue elevation of the surgical flap, osteotomy and dental sectioning in mandibular third molar extraction surgery promoted less edema in the early postoperative stages in mandibular third molar extractions despite the longer surgical duration.

## References

[1] Kirtiloğlu T, Bulut E, Sümer M, Cengiz I (2007). Comparison of 2 flap designs in the periodontal healing of second molars after fully impacted mandibular third molar extractions. J Oral Maxillofac Surg.

[2] Steed MB (2014). The indications for third-molar extractions. J Am Dent Assoc.

[3] Rosa AL, Carneiro MG, Lavrador MA, Novaes AB Jr (2002). Influence of flap design on periodontal healing of second molars after extraction of impacted mandibular third molars. Oral Surg Oral Med Oral Pathol Oral Radiol Endod.

[4] Pavlíková G, Foltán R, Horká M, Hanzelka T, Borunská H, Sedý J (2011). Piezosurgery in oral and maxillofacial surgery. Int J Oral Maxillofac Surg.

[5] Birkenfeld F, Becker ME, Harder S, Lucius R, Kern M (2012). Increased intraosseous temperature caused by ultrasonic devices during bone surgery and the influences of working pressure and cooling irrigation. Int J Oral Maxillofac Implants.

[6] Guillaume B, Gaudin C, Georgeault S, Mallet R, Basle MF, Chappard D (2009). Viability of osteocytes in bone autografts harvested for dental implantology. Biomed Mater.

[7] Labanca M, Azzola F, Vinci R, Rodella LF (2008). Piezoelectric surgery: Twenty years of use. Br J Oral Maxillofac Surg.

[8] Rahnama M, Czupkałło L, Czajkowski L, Grasza J, Wallner J (2013). The use of piezosurgery as an alternative method of minimally invasive surgery in the authors' experience. Wideochir Inne Tech Maloinwazyjne.

[9] Goyal M, Marya K, Jhamb A, Chawla S, Sonoo PR, Singh V (2012). Comparative evaluation of surgical outcome after removal of impacted mandibular third molars using a Piezotome or a conventional handpiece: a prospective study. Br J Oral Maxillofac Surg.

[10] Piersanti L, Dilorenzo M, Monaco G, Marchetti C (2014). Piezosurgery or conventional rotatory instruments for inferior third molar extractions?. J Oral Maxillofac Surg.

[11] Rullo R, Addabbo F, Papaccio G, D'Aquino R, Festa VM (2013). Piezoelectric device vs. conventional rotative instruments in impacted third molar surgery: relationships between surgical difficulty and postoperative pain with histological evaluations. J Craniomaxillofac Surg.

[12] World Medical Association Declaration of Helsinki: ethical principles for medical research involving human subjects (2013). World Medical Association. JAMA.

[13] Lima CJ, Silva LC, Melo MR, Santos JA, Santos TS (2012). Evaluation of the agreement by examiners according to classifications of third molars. Med Oral Patol Oral Cir Bucal.

[14] Sandhu A, Sandhu S, Kaur T (2010). Comparison of two different flap designs in the surgical removal of bilateral impacted mandibular third molars. Int J Oral Maxillofac Surg.

[15] Al-Moraissi EA, Elmansi YA, Al-Sharaee YA, Alrmali AE, Alkhutari AS (2016). Does the piezoelectric surgical technique produce fewer postoperative sequelae after lower third molar surgery than conventional rotary instruments?. A systematic review and meta analysis. Int J Oral Maxillofac Surg.

[16] Magesty RA, Galvão EL, de Castro Martins C, Dos Santos CRR, Falci SGM (2017). Rotary instrument or piezoelectric for the removal of third molars: a meta-analysis. J Maxillofac Oral Surg.

[17] Keyhan SO, Fallahi HR, Cheshmi B, Mokhtari S, Zandian D, Yousefi P (2019). Use of piezoelectric surgery and Er:YAG laser:which one is more effective during impacted third molar surgery?. Maxillofac Plast Reconstr Surg.

[18] Patil C, Jadhav A, K R, Bhola N, Borle RM, Mishra A (2019). "Piezosurgery vs bur in impacted mandibular third molar surgery: Evaluation of postoperative sequelae". J Oral Biol Craniofac Res.

[19] Menziletoglu D, Basturk F, Isik BK, Esen A (2020). A prospective split-mouth clinical study: comparison of piezosurgery and conventional rotary instruments in impacted third molar surgery. Oral Maxillofac Surg.

[20] de Freitas Silva L, Ribeiro de Carvalho Reis EN, Oliveira Souza BC, Egas LS, Aranega AM, Ponzoni D (2019). Alveolar repair after the use of piezosurgery in the removal of lower third molars: a prospective clinical, randomised, double-blind, split-mouth study. Br J Oral Maxillofac Surg.

